# Describing and Modeling Rough Composites Surfaces by Using Topological Data Analysis and Fractional Brownian Motion

**DOI:** 10.3390/polym15061449

**Published:** 2023-03-14

**Authors:** Antoine Runacher, Mohammad-Javad Kazemzadeh-Parsi, Daniele Di Lorenzo, Victor Champaney, Nicolas Hascoet, Amine Ammar, Francisco Chinesta

**Affiliations:** 1 PIMM Laboratory and ESI Group Chair, Arts et Metiers Institute of Technology, 151 Boulevard de Hopital, 75013 Paris, France; 2 IPC, 2 Rue Pierre et Marie Curie, 01100 Bellignat, France; 3 LAMPA Laboratory and ESI Group Chair, Arts et Metiers Institute of Technology, 2 bd du Ronceray, 49035 Angers, France; 4 ESI Group, 3 Rue Saarinen, 94150 Rungis, France

**Keywords:** topological data analysis—TDA, composite consolidation, rough surfaces, fractional Brownian surfaces

## Abstract

Many composite manufacturing processes employ the consolidation of pre-impregnated preforms. However, in order to obtain adequate performance of the formed part, intimate contact and molecular diffusion across the different composites’ preform layers must be ensured. The latter takes place as soon as the intimate contact occurs and the temperature remains high enough during the molecular reptation characteristic time. The former, in turn, depends on the applied compression force, the temperature and the composite rheology, which, during the processing, induce the flow of asperities, promoting the intimate contact. Thus, the initial roughness and its evolution during the process, become critical factors in the composite consolidation. Processing optimization and control are needed for an adequate model, enabling it to infer the consolidation degree from the material and process features. The parameters associated with the process are easily identifiable and measurable (e.g., temperature, compression force, process time, ⋯). The ones concerning the materials are also accessible; however, describing the surface roughness remains an issue. Usual statistical descriptors are too poor and, moreover, they are too far from the involved physics. The present paper focuses on the use of advanced descriptors out-performing usual statistical descriptors, in particular those based on the use of homology persistence (at the heart of the so-called topological data analysis—TDA), and their connection with fractional Brownian surfaces. The latter constitutes a performance surface generator able to represent the surface evolution all along the consolidation process, as the present paper emphasizes.

## 1. Introduction

**Figure 1:**
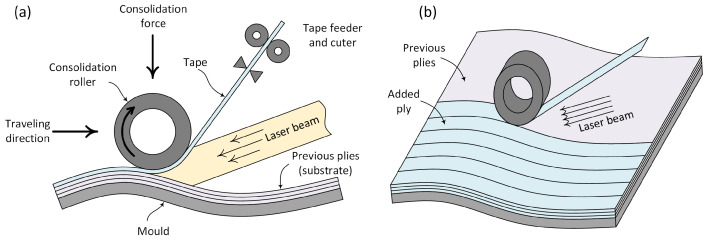
The automated tape placement (ATP) composite manufacturing: (**a**) physical phenomena; (**b**) processing.

Many manufacturing processes are based on the consolidation of composites’ pre-impregnated preforms that are available in the form of sheets or tapes. In both cases, the consolidation requires putting a new sheet (or tape) in contact with the one already laid, which now constitutes the so-called substrate [1]. If the temperature at the sheet–substrate contact level is high enough, the polymer viscosity becomes low enough to ensure the asperities flow under the externally applied pressure (from a press or from a roller in the case of automated tape laying as sketched in Figure 1). Thus, the initial asperities flow and spread, inducing the roughness smoothing and the increase in the intimate contact. As soon as the intimate contact occurs, at a temperature ensuring molecular mobility and during a time period long enough to ensure molecular reptation across the interface, consolidation is attained, and ideally bulk properties are recovered at the sheet interfaces.

Analysis and modeling of the roughness evolution are critical points for evaluating the quality of parts [2], to predict the remaining roughness that will result in a residual porosity in the composited laminate [3], and to evaluate tribological [4] or mechanical properties [5]. Other processes, such as machining, can induce noticeable roughness in the parts’ surfaces [6].

The design, optimization and control of the process involves description, modeling and simulation issues [7], revisited below.

### 1.1. Rough Surface Representation

Different approaches have been proposed to represent a rough surface in view of consolidation analyses. The simplest consists of representing the asperities as rectangular elements of a given height and width [8,9]. To consider more realistic representations, fractal surfaces have also been employed, where several characteristic dimensions are hierarchically present [10,11,12,13,14,15]. Finally, the most realistic option consists of acquiring the real surface itself, on which physics-based models can be applied. The use of wavelet representations is a valuable route, because of the fact that they favor a multi-resolution description, and that a simple description consisting of rectangles is easily manipulable when, for example, the Haar wavelet is considered [7].

All these descriptions enable surface representation for solving realistic physical models on them; however, as discussed later, they do not allow to easily perform surface comparisons or classifications. The latter, in general, require the use of adequate metrics, different to the usually considered Euclidean metrics. The use of dynamic time wrapping [16] could help in some cases, but it remains too macroscopic when different description scales co-exist (fine and coarse surface features), as is the case when considering micro and macro roughness.

When looking for more intrinsic descriptors, extracting roughness features that could serve to compare and classify surfaces, statistical descriptors [17,18,19,20,21] represent a first natural choice. However, in many cases such descriptors are quite far from the physics involved in the consolidation process, and, even if they are adequate for describing roughness from a geometrical point of view, they seem limited in representing the physics-based surface properties as well as the time-evolution during processing.

Indeed, there exist many advanced and richer descriptors of microstructures, such as the ones reported in a study by Torquato [22]. Richer descriptions should retain much more information but at the same time be more intrinsic than when one proceeds on the real surfaces, as previously mentioned. Thus, real surfaces could be expressed in alternative spaces by using well-experienced transformations, such as Fourier, discrete cosine transform, wavelets, ⋯, or by learning the appropriate transformation such as *Code2Vector* [23] performs, for instance.

Recently, alternative transformations emphasizing topological persistence, which are at the heart of the so-called TDA—topological data analysis (revisited later), are emerging and proving their value [24,25,26,27,28,29]. The so-called persistence diagram PD and its associated persistence image PI describe the topology of data in a very convenient way, such that surfaces exhibiting the same topology result in a similar PI. Persistence images, being defined in a vector space, can be easily compared by using adequate metrics (e.g., Euclidean, Wasserstein, ⋯) or manipulated by using standard machine learning technologies such as convolutional neural networks—CNN [30].

In [31] we proved that TDA can be used for classifying surfaces according to their topological content, out-performing previous studies making use of statistical descriptors [17], and proved it is possible to use that information to infer process-induced properties. By analyzing usual statistical roughness descriptors and TDA-based descriptors, respectively, in [17,31], the superiority of the latter was observed.

Another possible route consists of extracting the main modes representing these images by performing linear (principal component analysis—PCA) or nonlinear dimensionality reduction based on manifold learning [32], as successfully accomplished in [33], or by employing auto-encoders [34].

The main issue related to the use of statistical descriptors and transformation procedures is the possibility of generating synthetic surfaces according to these descriptors.

A valuable surface generator consists of exploiting the link between the random nature of roughness and Brownian motion. Generating time series, curves or surfaces from Brownian motion has been widely considered. However, Brownian motion seems, in some cases, too limited, in particular when processing occurs and the surface is strongly modified. In those cases, fractional Brownian motion seems a valuable route. Fractional motion is closely related to fractional diffusion, which, in turn, makes use of fractional calculus. Anomalous diffusion, also called non-Brownian diffusion or fractional diffusion, occurs when the mean square displacement scale with a non-integer power of time, this depending on the exponent value, defines surdiffusion (long-jumps) or subdiffusion (long-rest) [35].

Generating random surfaces by using fractional Brownian motion becomes a valuable route, and, even more interesting, this motion (as well as the resulting surfaces) is characterized from a single scalar, the so-called Hurst index. Fractional Brownian surfaces represent a timely research topic widely considered [36,37,38,39,40].

Thus, one could expect that modeling the Hurst index from some features, enables constructing models and makes it possible to reconstruct surfaces compatible with the features, a key route for material and process optimization and for processing control. This point represents the main goal of the present paper.

### 1.2. Original Contribution and Paper Outline

The present paper represents a step forward in the description of machine learning-based process modeling and process-induced properties.

Its main original contributions concern: (i) proving that TDA is able to discriminate fractional Brownian surfaces, the latter characterized by the Hurst index, in a very efficient manner, as soon as a regression model is developed for predicting the Hurst index from TDA-based persistence images; (ii) evaluating the effect of the composite compression on the persistence image, proving the ability of TDA to discriminate original and compressed surfaces; (iii) evidencing that the surface compression increases the Hurst index; and (iv) emphasizing the ability of fractional Brownian motion to generate synthetic surfaces with controlled topology, enabling further statistical analyses.

The paper is organized as follows. After the present introduction, the next section revisits the main methodologies employed in the present research work, in particular topological data analysis and fractional Brownian surface generation. Then, Section 3 addresses numerical analyses proving the ability of TDA to infer the Hurst index of fractional Brownian surfaces, as well as the evolution of topology and Hurst index during surface compression.

## 2. Methods

### 2.1. Topological Data Analysis

When TDA is applied on a time series, it transforms the time series into an image defined in a vector space, the so-called persistence image, that could be considered as a topological descriptor, enabling topological metrics to serve in comparing curves, or even to be employed as inputs in learned models.

For that purpose, and as explained in [31,41], when using a level-set-based filtration, neighboring local minima/local maxima are paired, leading to the so-called min–max pairs. Each min–max constitutes a point of the persistence diagram. The persistence diagram consists of a two-dimensional representation, where, on its horizontal axis, the minimum value of each data-pair is reported, while the associated maxima are displayed on the vertical axis. Because the maximum is always higher than the associated minimum, the axes of the persistence diagram are usually labeled as birth and death axes, respectively.

**Figure 2:**
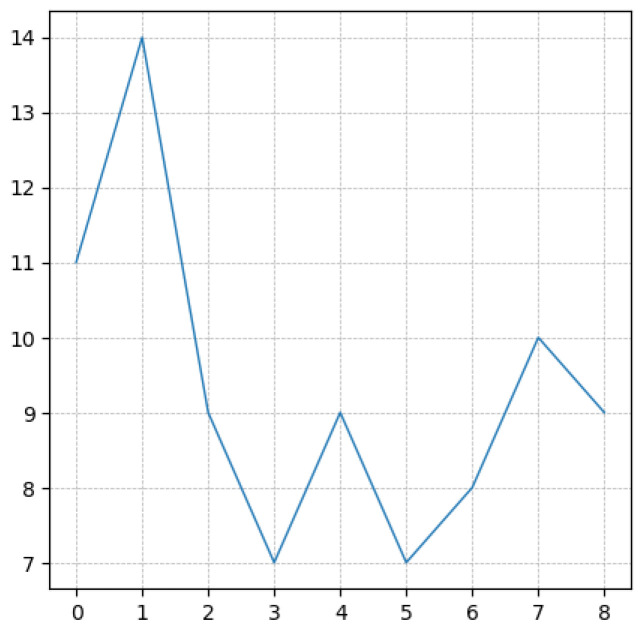
Random profile.

For the random profile depicted in Figure 2, (11,14), (7,9) and (9,10) represent the three neighboring local minima/local maxima.

**Figure 3:**
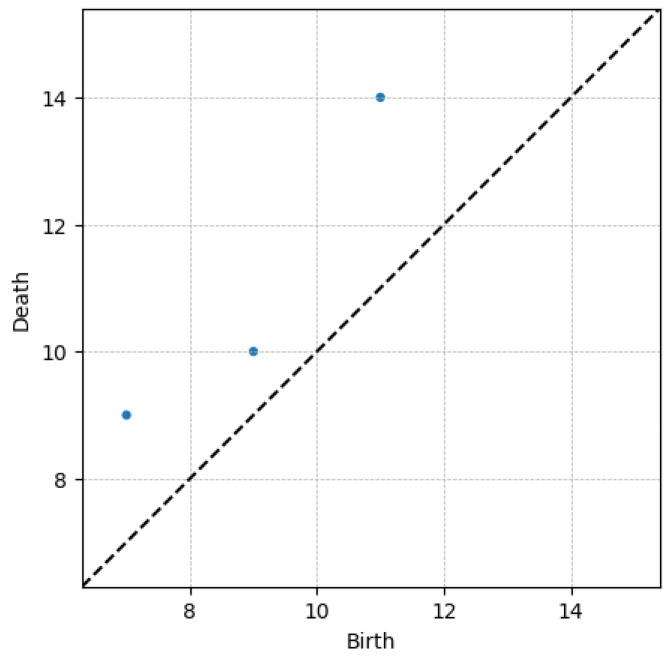
Typical persistence diagram PD.

Because of the nature of maximum and minimum (or birth and death) the points in the persistence diagram are located in the upper bisector defining the space y≥x, with *x* and *y* referring to birth and death (horizontal and vertical), respectively. The persistence diagram related to the profile depicted in Figure 2 is shown in Figure 3.

The higher the distance of a point to the diagonal x=y, the more persistent the topology of the considered point (maximum to minimum difference). Points located close to the diagonal contain a small persistent topology, which in many cases can be associated to noise (or finer physical scales).

Another important point concerns the invariance properties that the topology provides. If we consider a point on the persistence diagram depicted in Figure 3, for example the (11,14)-pair, this point is associated with local-minimum/local-maximum neighboring data points. All the pairs with a minimum value equal to 11, and the neighboring local maximum equal to 14, are associated with the referred (11,14)-pair, all of them represented by the same data-point in the persistence diagram, independently of their location on the time axis, or the time delay between the minimum and maximum constituting each pair.

Thus, two identical time series, but one of them translated in the time axis with respect to the second one, are represented by the same persistence diagram. Two time series, one of them consisting of a dilation of the other, have the same persistence diagram. These invariance properties are very important when the topology is more important than the location at which these topological events take place.

When addressing heat and momentum transfer on rough interfaces, TDA seems a very promising descriptor that extracts geometrical features closely related to the physics operating on it.

When topologies are close but not identical, their associated persistence diagrams consist of two set of points. To evaluate the distance between them, appropriate metrics must be employed for comparing the distance between both point clouds. Thus, the Wasserstein distance consists of a simple and efficient choice for measuring the distance between two persistence diagrams.

**Figure 4:**
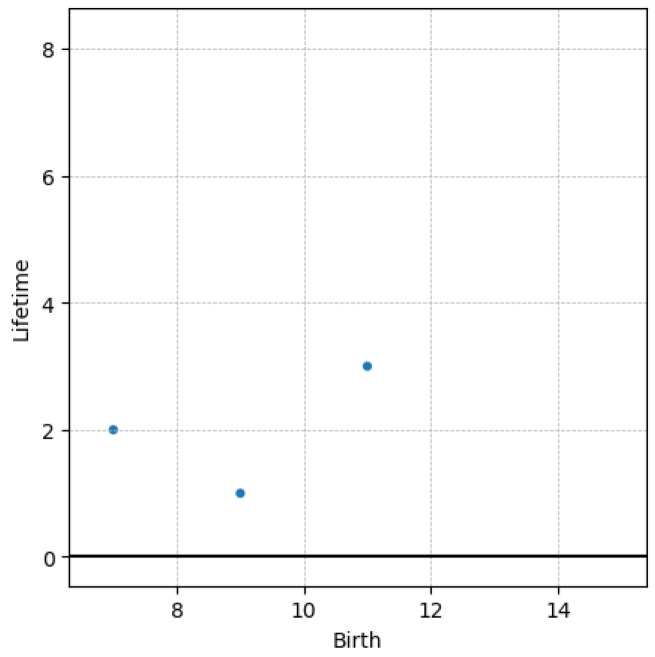
Lifetime diagram LT.

Another derived representation consists of the so-called persistence image. Prior to its introduction, an intermediate transformation is needed. Instead of representing birth–death, an equivalent representation consists of representing birth–lifetime, the latter defined as the difference between the death and the birth. The main advantage of using the lifetime diagram instead of the usual persistence diagram, is that in the former points fill the whole 2D space instead of only the upper bisector (y≥x). The lifetime diagram LT associated with the persistence diagram PD shown in Figure 3 is given in Figure 4.

Now, the last step consists of smoothing the point representation involved in the lifetime diagram, to transform a point into a 2D function. For that purpose, and as described in [31], points in the lifetime diagram are replaced by a Gaussian function centered at each point of the lifetime diagram LT.

Next, the so-called persistent image PI, defined in a vector space is constructed. For that purpose we consider a continuous piece-wise differentiable non-negative weight function w(x,y) and a bivariate normal distribution $g_{x,y}\left(u,v\right)$ centered at each point (x,y), from which a function ρ(u,v) can be defined at each point (u,v) in the lifetime domain [31]

(1) $$\rho \left(u,v\right)=\sum_{(x,y)\in \mathrm{LT}}w\left(x,y\right)\;g_{\left(x,y\right)}\left(u,v\right).$$

Now, the domain is partitioned in a series of non-overlapping subdomains covering it, the so-called pixels $P_{i}$, i=1,2,⋯, where function ρ(u,v) is averaged to define the *persistence image* PI, according to

(2) $$\mathrm{PI}\left(P_{i}\right)={\;\int \int }_{P_{i}}\rho \left(u,v\right)\;du\;dv.$$

**Figure 5:**
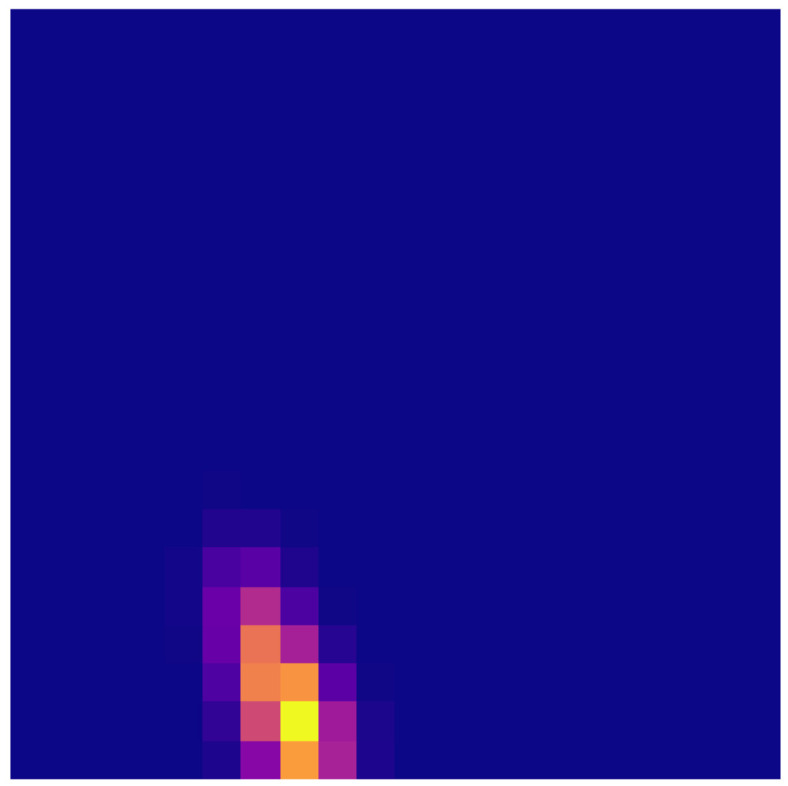
Typical persistence image.

A typical persistence image related to a profile larger than the one shown in Figure 2 is illustrated in Figure 5. Now, this being a quite standard image, one is tempted to use it as input in learning procedures, in particular those making use of convolutional neural networks—CNN [33], even if many other options exist. Other possibilities consist of applying dimensionality reduction, either linear (e.g., PCA) or nonlinear (e.g., auto-encoders), to facilitate the neural network training, that now makes use of smaller input data, or of applying the weights of PCA modes when using the PCA or the data mapped into the latent reduced space when considering auto-encoders.

### 2.2. From Brownian Diffusion to Anomalous Diffusion

This section revisits the derivation of Brownian diffusion and its connection with Brownian motion, viewed as a random walk. Then, in the case of anomalous diffusion, fractional Brownian motion will lead to the so-called fractional Brownian trajectories and surfaces.

#### 2.2.1. Brownian Diffusion

Following Einstein’s works, the particle displacement Δ in the unbounded one-dimensional axis *x* is represented by a random variable whose probability density reads ϕ(Δ).

The particle’s balance considers both, the local time evolution

(3) $$\rho \left(x,t+\tau \right)=\rho \left(x,t\right)+\frac{\partial \rho (x,t)}{\partial t}\tau +\Theta \left(\tau^{2}\right),$$

and the transport in space

(4) $$\rho \left(x,t+\tau \right)=\int_{R}\rho \left(x+\Delta ,t\right)\phi \left(\Delta \right)d\Delta .$$

Developing ρ(x+Δ,t)

(5) $$\rho \left(x+\Delta ,t\right)=\rho \left(x,t\right)+\frac{\partial \rho (x,t)}{\partial x}\Delta +\frac{1}{2}\frac{\partial^{2}\rho \left(x,t\right)}{\partial x^{2}}\Delta^{2}+\Theta \left(\Delta^{3}\right)$$

and taking into account the normality $\int_{R}\phi \left(\Delta \right)d\Delta =1$ and symmetry $\int_{R}\Delta \;\phi \left(\Delta \right)d\Delta =0$ conditions, the final result is

(6) $$\rho \left(x,t\right)+\frac{\partial \rho (x,t)}{\partial t}\tau =\rho \left(x,t\right)+\frac{1}{2}\frac{\partial^{2}\rho \left(x,t\right)}{\partial x^{2}}\int_{R}\Delta^{2}\phi \left(\Delta \right)d\Delta ,$$

or

(7) $$\frac{\partial \rho (x,t)}{\partial t}\tau =D\frac{\partial^{2}\rho \left(x,t\right)}{\partial x^{2}},$$

with

(8) $$D=\frac{1}{2\tau }\int_{R}\Delta^{2}\phi \left(\Delta \right)d\Delta .$$

The integration of the diffusion equation from the localized (at x=0) initial condition $\rho \left(x,t=0\right)=\delta_{0}\left(x\right)$, leads to the Gaussian distribution

(9) $$\rho \left(x,t\right)=\frac{1}{\sqrt{4\pi Dt}}e^{-\frac{x^{2}}{4Dt}},$$

whose main square displacement scales linearly with the time

(10) $$\langle x^{2}\rangle =2Dt.$$

#### 2.2.2. Brownian Motion

The diffusion equation can be also derived from a random walk. For illustrating the derivation, we consider a grid on the x-axis, with step Δx being the time step Δt. At each time step, each particle is assumed to jump to one of its two neighboring sites with the same probability.

Thus, the balance at the *j*-site (grid node) reads

(11) $$W_{j}\left(t+\Delta t\right)=\frac{1}{2}W_{j+1}\left(t\right)+\frac{1}{2}W_{j-1}\left(t\right),$$

where $W_{j}\left(t\right)$ is the probability of having the particle at site *j* at time *t*.

By developing $W_{i}\left(t+\Delta t\right)$, $W_{j+1}\left(t\right)$ and $W_{j-1}\left(t\right)$ according to

(12) $$\left\{\begin{array}{c} W_{i}\left(t+\Delta t\right)=W_{j}\left(t\right)+{\left.\frac{\partial W_{j}\left(t\right)}{\partial t}\right|}_{t}\Delta t+\Theta \left(\Delta t^{2}\right)\\ W_{j+1}\left(t\right)=W_{j}\left(t\right)+{\left.\frac{\partial W(t)}{\partial x}\right|}_{j}\Delta x+\frac{1}{2}{\left.\frac{\partial^{2}W\left(t\right)}{\partial x^{2}}\right|}_{j}\Delta x^{2}+\Theta \left(\Delta x^{3}\right)\\ W_{j-1}\left(t\right)=W_{j}\left(t\right)-{\left.\frac{\partial W(t)}{\partial x}\right|}_{j}\Delta x+\frac{1}{2}{\left.\frac{\partial^{2}W\left(t\right)}{\partial x^{2}}\right|}_{j}\Delta x^{2}-\Theta \left(\Delta x^{3}\right) \end{array}\right.,$$

the balance results in the usual diffusion equation

(13) $$\frac{\partial W}{\partial t}=D\frac{\partial^{2}W}{\partial x^{2}},$$

with *D* defined in the limit of infinitesimal Δx and Δt by

(14) $$D=\frac{\Delta x^{2}}{2\Delta t}.$$

### 2.3. Fractional Brownian Motion

The equivalence between Brownian diffusion and Brownian motion motivates the modeling of anomalous diffusion (which results in the fractional diffusion equation [35]) by using fractional Brownian motion.

In fractional Brownian motion, also known as fractal Brownian motion, the increments are not independent, in contrast to Brownian motion.

For that purpose, we consider the continuous Gaussian process $B_{H}\left(t\right)$ (the index ${•}_{H}$ refers to the Hurst index), with $B_{H}\left(t=0\right)=0$, having a null expectation at each time, and defined by the covariance

(15) $$E\left(B_{H}\left(r\right),B_{H}\left(s\right)\right)=\frac{1}{2}\left({\left|r\right|}^{2H}+{\left|s\right|}^{2H}-{\left|r-s\right|}^{2H}\right),$$

with H=0.5 in the case of Brownian motion.

For generating a random trajectory we consider a set of time instants $t_{1},\;t_{2},\cdots ,t_{n}$ and construct the matrix R (symmetric positive–definite), whose component $R_{ij}$ given by

(16) $$R_{ij}=\frac{1}{2}\left(|t_{i}{|}^{2H}+|t_{j}{|}^{2H}-{\left|t_{i}-t_{j}\right|}^{2H}\right).$$

The singular value decomposition—SVD of R writes $R=VDV^{T}$. Thus, we can define the matrix $S=VD^{1/2}V^{T}$.

Now, we generate *n* values following a standard Gaussian distribution (zero mean and unit variance), grouped in vector G. Finally, this results in the associated fractional values in vector $F_{H}$:

(17) $$F_{H}=SG.$$

## 3. Numerical Tests

### 3.1. Topological Data Analysis

Different pre-impregnated composite sheets, delivered by the same provider and prepared with the same materials and under the same process conditions, were analyzed, to check the existence of topological variability on their respective surfaces. A Bruker optical profilometer was used to extract the surface roughness profiles along different directions on the sheet surfaces, in particular along the direction of fibers, along the perpendicular direction to those fibers, and along long fibers that constitute the pre-impregnated composite sheets reinforcement.

**Figure 6:**
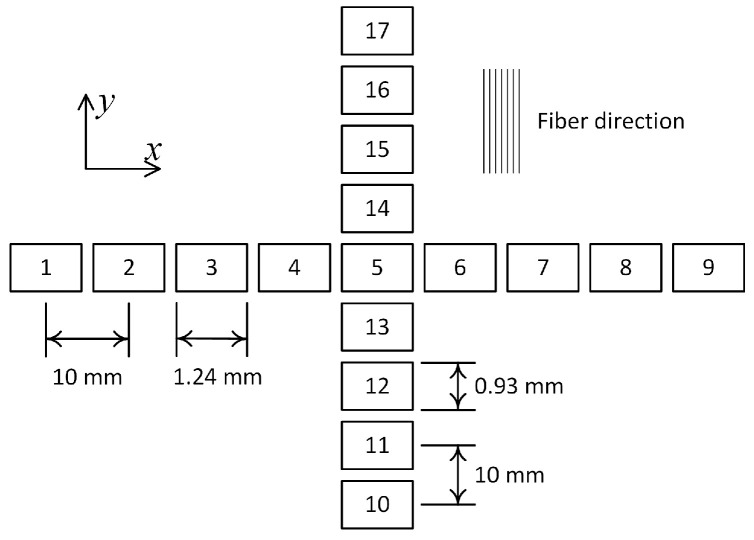
Measurement locations on the composite sheets’ surfaces.

Roughness profiles were measured at 17 different locations on both surfaces of each composite sheet. The locations of those measured zones and their dimensions are illustrated in Figure 6, where the fibers’ orientation is also indicated.

**Figure 7:**
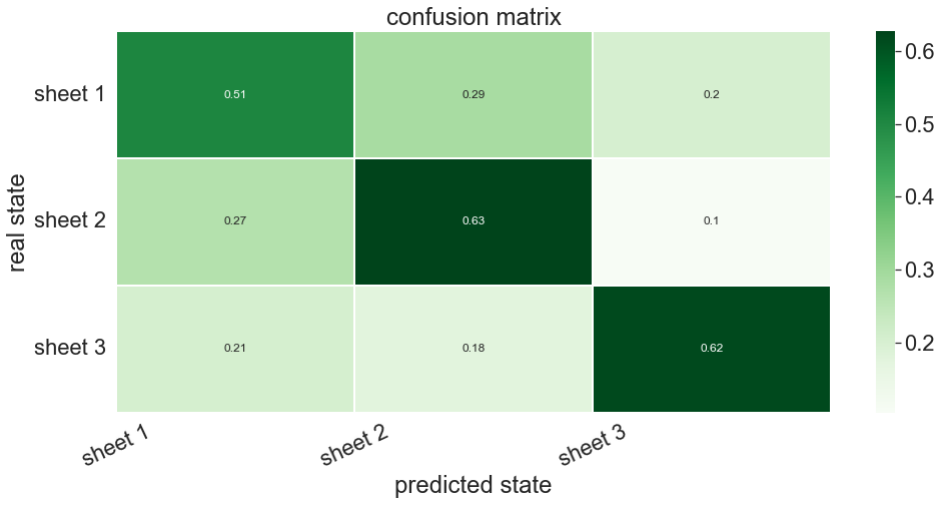
Confusion matrix related to the profiles of the three composite sheets.

The persistence images associated with the different profiles measured along a fiber’s direction and along its perpendicular direction, were obtained. A supervised random forest classifier involving 400 trees was trained to discriminate the profiles related to each of the three available composite sheets. A total of 80% of the 1020 available profiles was considered in the training, whereas the remaining 20% served to test the trained classifier performances. Figure 7 shows the associated confusion matrix related to the test set. This figure proves that the three sheet surfaces exhibited similar roughness from a topological viewpoint, and consequently the trained classifier failed to discriminate to which sheet each profile in the test set belongs.

Different classifiers were tested, all them exhibiting similar performances. To conclude on the reasons of the poor classifier performance, different persistence images extracted from the three sheets were analyzed and it was noticed that the differences between the images coming from the same sheets were similar to the differences between the images of different sheets. Thus, it can be concluded that the poor classification capabilities are due to the nature of the surfaces, instead of being a consequence of the classifiers’ performance. In these analyses, the Wasserstein distance was considered for quantifying the differences between the topological description of the surfaces.

However, during composite processing, the sheet surfaces will experience a compression affecting the roughness. Even if, as commented, no difference is expected between the different sheets, a difference is expected concerning the roughness topology before and after compression. To prove that discrimination ability, a sample of each sheet was heated to 200 degrees Celsius and compressed by using a pneumatic press, during 5 min, for ensuring the flow of surface asperities.

**Figure 8:**
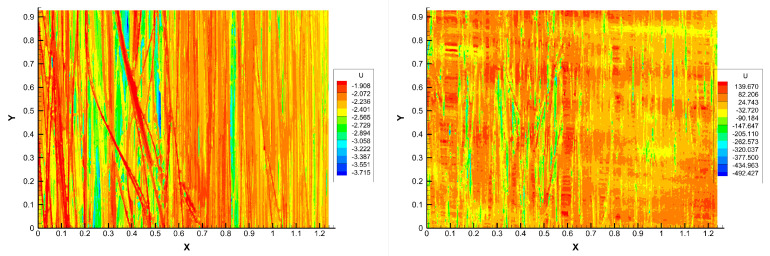
Surface topography before (**left**) and after (**right**) compression.

Figure 8 compares two characteristic surface topographies before and after compression, from which a noticeably higher smoothness can be seen on the compressed surface. Then, different rough profiles were measured by again using the optical profilometer in both directions (with respect to the fiber orientation).

As the roughness is significantly higher in the perpendicular direction to the fiber orientation, our analyses mainly focus on profiles along that perpendicular direction.

**Figure 9:**
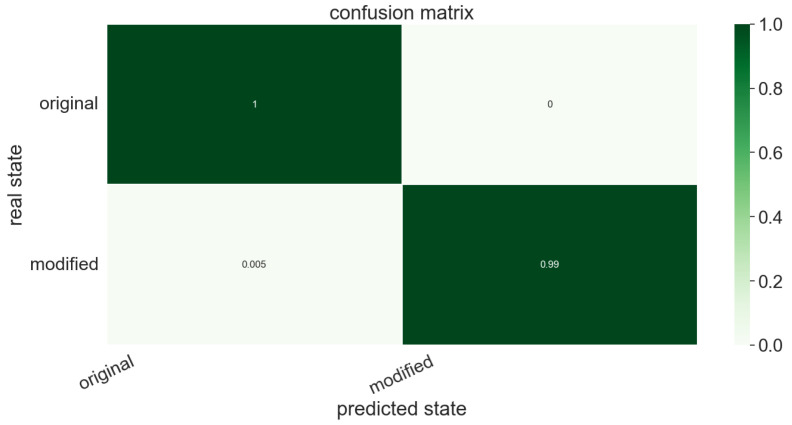
Confusion matrix related to the profiles of the three composite sheets.

Figure 9 shows the confusion matrix associated with a random forest classifier (involving 400 trees), trained from 1016 profiles and tested on 254 profiles that were excluded from the training set. It can be concluded that TDA perfectly discriminates original and compressed surfaces.

**Figure 10:**
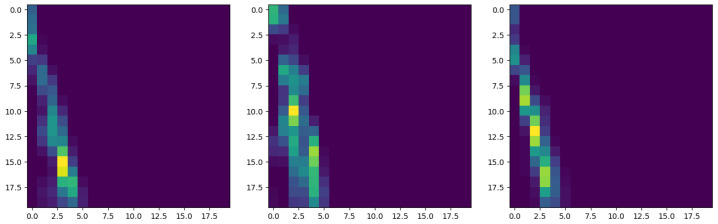
Persistence images related to three roughness profiles, each extracted from one of the three composite sheets.

To better visualize the roughness-topology change induced by the compression, Figure 10 shows three persistence images, one extracted from each of the three composite sheets along the perpendicular direction to the long fibers, where no major differences are noticed between them.

These figures also reveal an almost mono-disperse topological content, which explains the localized pattern present in the persistence images.

**Figure 11:**
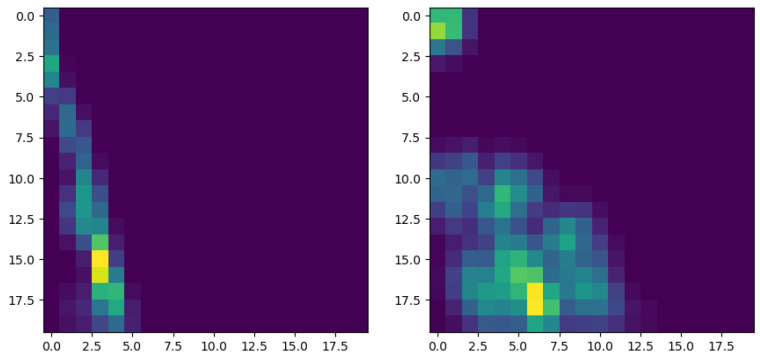
Characteristic persistence images before (**left**) and after (**right**) compression.

On the other side, Figure 11 compares the roughness topologies before and after compression, where a significant difference can clearly be noticed; the topology is more poly-disperse after compression. On the original surface the asperities’ height seems quite mono-disperse, with the associated localization in the persistence image. When compressing the surface, the highest asperities start to be compressed and the asperities’ height starts exhibiting a larger spectrum, with the associated effect on the persistence image.

### 3.2. Fractional Brownian Surfaces

This section aims at generating different roughness profiles by using fractional Brownian motion, characterized by different Hurst indexes. Then, a classifier is trained with the associated TDA surfaces, and the classifier’s performance is tested on the surfaces composing the test set.

**Figure 12:**
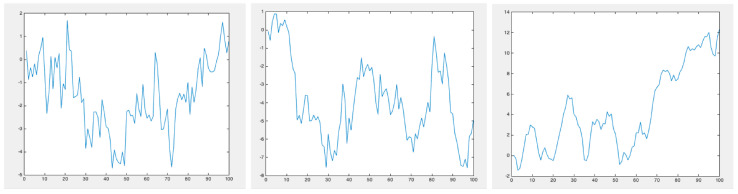
Profiles generated by a fractional Brownian motion characterized by H=0.25 (**left**), H=0.5 (**center**) and H=0.75 (**right**).

Figure 12 shows three surfaces generated from a fractional Brownian motion with H=0.25, H=0.5 and H=0.75. No major differences are noticeable at first glance; however, a topological footprint is expected to exist, enabling an efficient classification.

**Figure 13:**
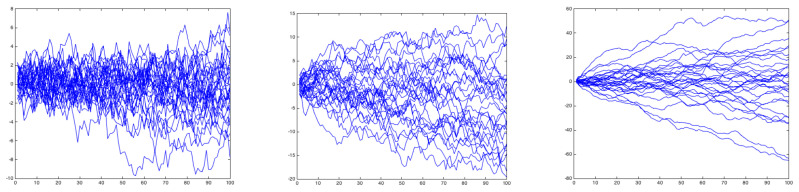
Population of 30 profiles generated by a fractional Brownian motion characterized by H=0.25 (**left**), H=0.5 (**center**) and H=0.75 (**right**).

**Figure 14:**
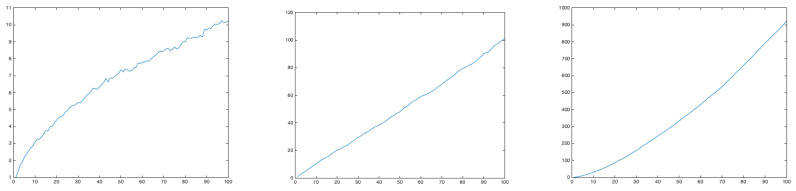
Mean square displacement evolution for the population of profiles generated by a fractional Brownian motion characterized by H=0.25 (**left**), H=0.5 (**center**) and H=0.75 (**right**).

To prove the Brownian behavior of profiles generated with H=0.5, and the anomalous diffusion for H<0.5 and H>0.5, we generated 1000 trajectories and evaluated their mean square displacement evolution. Figure 13 superposes 30 different profiles generated with H=0.25, H=0.5 and H=0.75. Figure 14 depicts the evolution of the mean square displacement. As expected, it can be noticed that for H=0.5 (Brownian motion) the mean square displacement evolves linearly.

Now, the persistence images associated with each profile are obtained, and a part of them (2400 images) used for training a random forest classifier (involving 400 trees), which is then applied for classifying 600 persistence images composing the test set.

**Figure 15:**
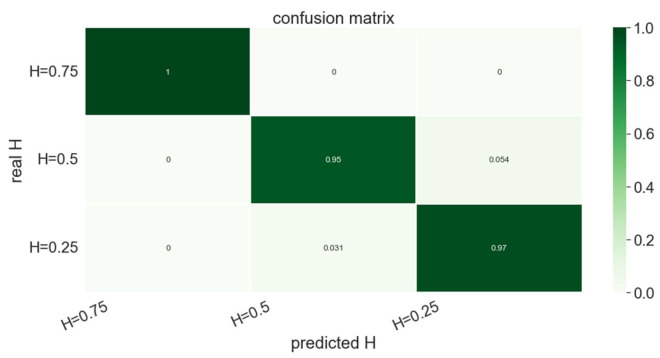
Confusion matrix related to the trained classifier applied to the three fractional Brownian surfaces characterized by H=0.25, H=0.5 and H=0.75.

The confusion matrix associated with the classifier, again a random forest involving 400 trees, trained from 8000 images, applied to the test set (composed of 2000 images) is shown in Figure 15 and proves the ability of the TDA to capture the different data topologies associated with the different Hurst indexes.

**Figure 16:**
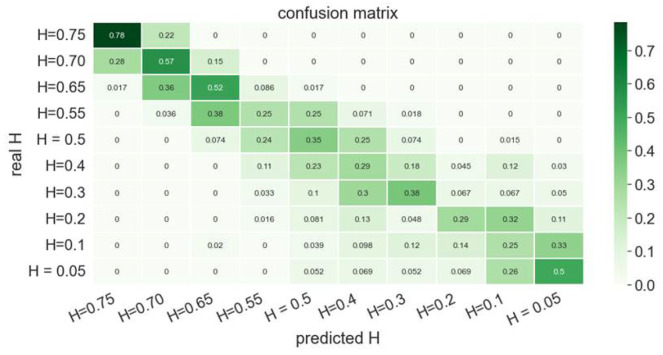
Confusion matrix related to the trained classifier applied to ten fractional Brownian surfaces.

In order to prove the proposed model’s robustness, the procedure was repeated, but now considered 10 different Hurst indexes. Again, the confusion matrix associated with the trained classifier, shown in Figure 16, proves the TDA’s robustness for representing fractional Brownian surfaces.

### 3.3. Hurst Index Evolution during the Surface Compression

To better understand the effect of surface compression on the Hurst index, we considered many Brownian surfaces generated by considering H=0.5. Then, we removed all the positive peaks, that is, the positive part of all the profiles was removed and replaced by a null value, for emulating the flatness induced by the compression. The asperities’ flattening is compulsory for consolidation to occur, because of the fact that molecular reptation needs the intimate contact that flatness facilitates. Then, a regressor (random forest involving 400 trees) operating on the persistence images of 4800 profiles, with Hurst indexes ranging in the interval (0.05,0.75), and tested on the 1200 images composing the test set, was applied to the persistence images associated with flattened profiles. It was observed that the inferred Hurst index ranged in the interval (0.55,0.6), proving that, as expected, higher correlations are induced in the data representing the flattened profiles.

**Figure 17:**
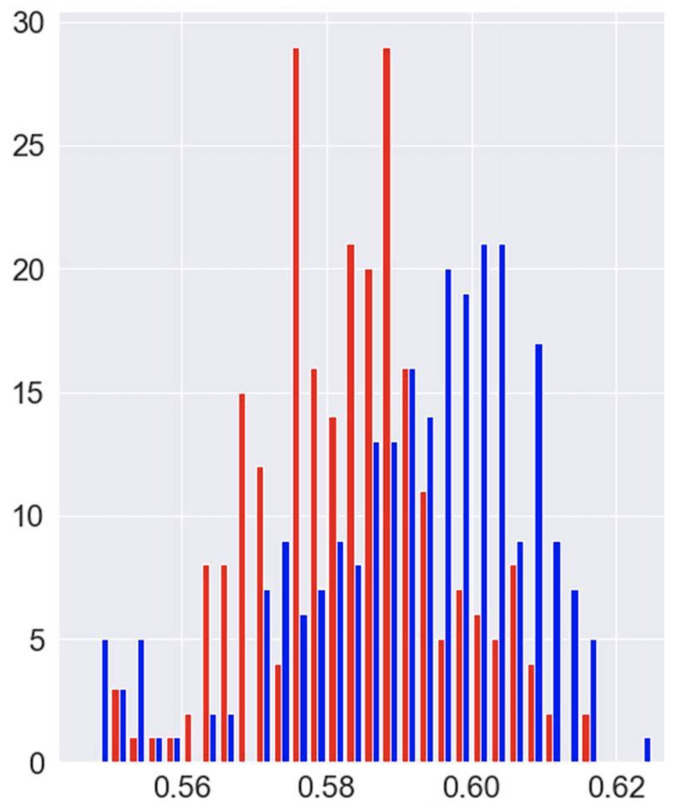
Inferred Hurst index histograms for 250 non-compressed (red) and 250 compressed (blue) profiles.

Now, the same rationale was applied to the real composites’ profiles, for comparing the Hurst index distribution before and after compression. For that purpose 250 non-compressed and other 250 compressed profiles, randomly chosen from the 1270 available, were analyzed, and their Hurst indexes inferred by using the trained regressor just introduced. The histograms related to the inferred Hurst indexes are compared in Figure 17, evidencing that the compression induces a noticeable increase in the Hurst index distribution.

## 4. Conclusions

The present paper first proved the ability of topological data analysis to quantify the roughness evolution during composite processing involving compression, and at the same time characterize the roughness of pre-impregnated composite sheets.

Then, the link between TDA and fractional Brownian surfaces was evidenced. TDA enables classifying those surfaces according to their associated Hurst indexes.

Finally, compressed and non-compressed surfaces were characterized using a Hurst index evolution, which could represent a valuable descriptor in processing optimization and control.

## Data Availability

Data is available upon request.
